# Study design and participant characteristics of a randomized controlled trial of directly administered antiretroviral therapy in opioid treatment programs

**DOI:** 10.1186/1471-2334-11-45

**Published:** 2011-02-15

**Authors:** Bernadette Anna Mullen, Katie Cook, Richard D Moore, Cynthia Rand, Noya Galai, Mary E McCaul, Sheldon Glass, Krisann K Oursler, Gregory M Lucas

**Affiliations:** 1 Division of Infectious Diseases, Department of Medicine, Johns Hopkins University School of Medicine, Baltimore, Maryland, USA; 2 Division of General Internal Medicine, Department of Medicine, Johns Hopkins University School of Medicine, Baltimore, Maryland, USA; 3 Division of Pulmonary and Critical Care, Department of Medicine, Johns Hopkins University School of Medicine, Baltimore, Maryland, USA; 4 Department of Epidemiology, Johns Hopkins University, Bloomberg School of Public Health, Baltimore, Maryland, USA and the Department of Statistics, University of Haifa, Haifa, Israel; 5 Department of Psychiatry and Behavioral Sciences, Johns Hopkins University School of Medicine, Baltimore, Maryland, USA; 6 Department of Medicine, University of Maryland School of Medicine, Baltimore, Maryland, USA

## Abstract

**Background:**

HIV-infected drug users are at higher risk of non-adherence and poor treatment outcomes than HIV-infected non-drug users. Prior work from our group and others suggests that directly administered antiretroviral therapy (DAART) delivered in opioid treatment programs (OTPs) may increase rates of viral suppression.

**Methods/Design:**

We are conducting a randomized trial comparing DAART to self-administered therapy (SAT) in 5 OTPs in Baltimore, Maryland. Participants and investigators are aware of treatment assignments. The DAART intervention is 12 months. The primary outcome is HIV RNA < 50 copies/mL at 3, 6, and 12 months. To assess persistence of any study arm differences that emerge during the active intervention, we are conducting an 18-month visit (6 months after the intervention concludes). We are collecting electronic adherence data for 2 months in both study arms. Of 457 individuals screened, a total of 107 participants were enrolled, with 56 and 51 randomly assigned to DAART and SAT, respectively. Participants were predominantly African American, approximately half were women, and the median age was 47 years. Active use of cocaine and other drugs was common at baseline. HIV disease stage was advanced in most participants. The median CD4 count at enrollment was 207 cells/mm^3^, 66 (62%) had a history of an AIDS-defining opportunistic condition, and 21 (20%) were antiretroviral naïve.

**Conclusions:**

This paper describes the rationale, methods, and baseline characteristics of subjects enrolled in a randomized clinical trial comparing DAART to SAT in opioid treatment programs.

**Trial Registration:**

ClinicalTrials.gov: NCT00279110

## Background

Founded on the successful model of directly observed therapy for tuberculosis [1], directly administered antiretroviral therapy (DAART) for HIV-infected individuals has been evaluated in several contexts [2-4]. To date, trials have yielded mixed results regarding the efficacy of DAART for increasing adherence and viral suppression rates compared to self-administered therapy (SAT). A meta-analysis of randomized trials found no benefit for DAART relative to SAT for viral load suppression [2]. In contrast, a second meta-analysis, which included both randomized and non-randomized comparative studies, found DAART to be statistically significantly associated with a higher likelihood of viral suppression and larger CD4 cell increases compared to SAT [3]. Both meta-analyses reported trends for greater DAART benefit in studies conducted in drug-using compared to non-drug-using populations.

Medication assisted therapy with methadone or buprenorphine in opioid treatment programs (OTPs) is effective treatment for opioid addiction, and entails frequent contact with patients that may facilitate DAART [5,6]. Based on developmental work at our center [7], we hypothesized that providing DAART to HIV-infected individuals in OTPs would be effective and sustainable. To address this question we designed a randomized controlled trial of DAART compared with SAT. In this paper we describe the rationale, methods, and baseline characteristics of subjects enrolled in our study.

## Methods

### Design

The study is a randomized, non-blinded trial comparing DAART and SAT in HIV-infected participants who are receiving medication assisted treatment at 1 of 5 OTPs in Baltimore, Maryland. In the DAART intervention, study assistants observe morning doses of antiretroviral therapy (ART) on weekdays when participants attend the OTP; other doses are self-administered. Participants in the control arm self-administer all doses. The primary outcome is viral load suppression, measured at 3, 6, and 12 months. We provide DAART for up to 12 months, after which participants assigned to this arm convert to self-administration. We follow participants to 18 months to assess the potential persistence of any benefits realized during the intervention period.

### Setting

We enrolled participants from 5 OTPs in Baltimore between May 2006 and May 2010. Initially, the study began with three sites (1, 2, and 3). However, we discontinued recruitment at site 3 due to slow enrollment and replaced it with sites 4 and 5 in August 2007 and August 2008, respectively. Three programs were hospital-affiliated OTPs (sites 1, 3, and 5), and two were independent programs (sites 2 and 4). The OTP censuses ranged from 153 to 1007. All sites provided methadone maintenance therapy, and site 1 also provided buprenorphine maintenance therapy. Sites 1 and 5 had on-site HIV care available throughout the study, sites 2 and 3 had on-site HIV care available for part of the study period, and site 4 did not have on-site HIV-care services.

### Eligibility

Individuals were eligible for the study if they were 18 years of age or older, HIV seropositive, had received maintenance therapy with methadone or buprenorphine for > 3 weeks with no plans to discontinue, and had an identified HIV provider and active insurance coverage for ART. Moreover, to be included in the study, we required participants to be ART experienced or (if treatment naïve) eligible for ART according to March 23, 2004 Department of Health and Human Services guidelines for treatment [8], which included history of opportunistic condition, HIV-related symptoms, CD4 count < 350 cells/mm^3^, or HIV RNA > 55,000 copies/mL. ART regimens were prescribed by HIV providers prior to randomization and had to include ≥ 3 drugs, including a protease inhibitor, non-nucleoside reverse transcriptase inhibitor, abacavir, or integrase inhibitor. Additionally, we required verbal agreement to participate from participants' HIV medical providers. Exclusion criteria included stable ART use with HIV RNA < 500 copies/mL, ART dosed more frequently than twice daily, use of liquid antiretroviral preparations, participation in another HIV adherence program in which medication is directly administered, or fewer than 1.5 'active' drugs in the specified regimen - as predicted by prior genotypic resistance tests (if available) and mutation interpretation guidelines [9].

### Participant Recruitment

Research assistants, employed full-time at the sites and familiar with the OTP clients, were responsible for recruitment and initial screening of participants. We posted study flyers at all sites and made regular presentations to counselors, intake specialists, and administrators to describe the purpose of the study and request referral of HIV-infected clients for screening. Additionally, we facilitated on-site HIV counseling and testing services at the sites to improve access to testing and to identify HIV-infected persons who were not aware of their status. The research assistants and study coordinator obtained and reviewed medical records and contacted HIV care providers to obtain their agreement to participate.

### Human Subjects Protection

The study was approved by the Johns Hopkins Medicine Institutional Review Board, the University of Maryland Baltimore Institutional Review Board, and the Veterans Administration Research and Development Committee. Participants provided written informed consent, and were encouraged to take the consent document home to discuss with family or medical providers prior to joining the study. We also asked participants to sign releases for medical and substance abuse treatment records to permit confirmation of study eligibility and to record outcome data during the trial. To protect confidentiality, unique study identification numbers were used on all case report forms and clinical samples. We stored hard copies of study records in locked filing cabinets inside locked offices. We store electronic data on a password-protected laptop computer that was backed-up each night to a secure server. We obtained a Certificate of Confidentiality from the National Institute on Drug Abuse to further protect sensitive participant data.

### Randomization

To prevent knowledge of treatment assignment from influencing the selection of antiretroviral drugs, we required HIV providers to prescribe ART regimens prior to randomization. Prior to the study, one of the investigators (GML) generated random treatment assignments to DAART and SAT in a 1:1 ratio, stratified by study site and ART exposure at baseline (naïve or experienced), using a commercial statistical software package. Assignments were generated in blocks that randomly varied in size between 2 and 8. The treatment assignment list was incorporated into a Microsoft Access-based program that revealed individual assignments sequentially as new participants were enrolled. When enrollment criteria were fulfilled for a new participant, the study coordinator obtained the treatment assignment by activating the "randomize" function in the computer program. The assignment list was password protected and was not accessible to the research coordinator or research assistants, apart from revealing sequential treatment assignments at the times of randomization.

### Participant Follow-up

We conducted study visits at baseline, 3, 6, 12, and 18 months. Study visits were conducted at OTPs and included an update of contact information, a face-to-face interview, and collection of blood and urine specimens. We measured CD4 cell counts (flow cytometry) and HIV RNA levels (AMPLICOR HIV-1 Monitor Test, version 1.5, Roche Diagnostics, Basel, Switzerland) from blood. We stored plasma samples from baseline and follow-up visits so that acquisition of drug resistance during the study period can be assessed. Urine samples were screened for methadone, opiates, cocaine, oxycodone, and benzodiazepines by enzyme immunoassay. Results of urine drug screens were not released to anyone outside of the study without written consent from participants.

The interviews addressed demographic and socioeconomic information, perceived availability of social support, depressive symptoms (Center for Epidemiologic Studies Depression Scale [CESD] short form [10]), anxiety symptoms (anxiety subscale of Brief Symptom Inventory [11]), self-reported ART adherence (3-day and 2-week recall of missed doses [12]), alcohol use (Alcohol Use Disorders Identification Test [13]), drug use (Johns Hopkins HIV Clinical Cohort Instrument [12]), health-related quality of life (visual analogue scale), and emergency department visits and hospitalizations in the prior 3 months.

The study coordinator conducted all study visits. This was done to both standardize data collection and to foster candid responses from participants about medication adherence, substance use, and other sensitive issues. The study coordinator had no role in participants' substance abuse treatment or in delivery of the DAART intervention, which was managed by research assistants at the sites. Additionally, study staff abstracted data from participants' OTP substance abuse treatment records and from HIV clinic records every 3 months, including changes in methadone or buprenorphine dose, urine drug test results, CD4 cell counts, HIV RNA levels, opportunistic conditions, and changes to ART. We made concerted efforts to maintain contact with participants (particularly if they left the OTP) and made arrangements for study visits to be completed in another venue if preferred. We updated telephone and mailing address contact information at each encounter and also collected contact information for relatives that would be likely to know participants' whereabouts. We reimbursed participants for completing study visits.

### DAART Arm

Two specialty pharmacies packaged medications for the DAART arm in single-dose clear plastic bags that were labeled with medication and dosing information as specified in Maryland State regulations. When participants attended the OTP for methadone or buprenorphine they went to a private office where a research assistant or a methadone nurse observed them take an ART dose. While other investigators have administered ART and methadone simultaneously from OTP dosing windows [6,14], participants in our pilot project found that this approach was stressful and jeopardized their confidentiality [7]. We provided participants with take-home ART doses (packaged identically to observed doses) for evenings (when required by a twice-daily dosing schedule), weekends, holidays, and weekdays when participants did not have to attend the OTP (i.e., methadone take-home days). We maintained close contact with the medical providers and asked them to notify us promptly of changes to the ART regimen and to send new prescriptions to us by facsimile so that medication could be prepared for observed dosing. DAART participants were also supplied with three days of "emergency doses" to use if they did not attend the OTP as scheduled. Emergency doses are replenished as needed.

### SAT Arm

SAT participants self-administered all ART doses. We did not provide adherence counseling, coaching, or feedback to SAT participants. SAT participants were free to engage in adherence programs offered by their HIV clinics.

### Safety Monitoring

We alerted patients and HIV medical providers about potential drug interactions between methadone and antiretroviral drugs that were prescribed. In particular, we assured that participants and providers were aware of the potential for non-nucleoside reverse transcriptase inhibitors to increase the metabolism of methadone, which can cause clinically significant opioid withdrawal symptoms [15,16]. We also notified participants' counselors and OTP clinicians so that methadone doses could be adjusted in a timely fashion if needed.

### Electronic Adherence Monitoring

We used MEMS VI Track Caps (Aardex Group, Zug, Switzerland) for electronic adherence monitoring (EAM) for the first 2 months of the study in both arms. Because these devices are not compatible with pill-boxes, the EAM substudy was not required if participants preferred to use pill boxes. We selected one medication from each participant's ART regimen for EAM according to the following selection criteria hierarchy: 1) medication dosed twice daily, 2) combination preparation, containing two or three antiretroviral drugs, 3) protease inhibitor (excluding ritonavir if only used for pharmacokinetic boosting), or 4) non-nucleoside reverse transcriptase inhibitor. Research assistants met with participants to initiate EAM when the new regimen was started. Research assistants instructed participants on the use of EAM devices (with verbal and written instructions), activated new EAM caps, completed quality control protocols, and placed caps on medication bottles. Participants returned for EAM visits at approximately 10 days, 4 weeks, and 8 weeks. At the each of these visits, research assistants downloaded data from the EAM devices, completed a debriefing form to identify non-adherence with use of the devices, reviewed instructions for proper use of EAM devices, performed quality control checks, counted leftover pills, and refilled the monitored medication with a new 30-day supply if needed (i.e., at 4-week visit). Research assistants did not discuss EAM data or provide feedback to participants. We reimbursed participants for attending EAM visits and returning EAM devices.

We modified the procedures described above for participants assigned to DAART. As with SAT participants, DAART participants took home an EAM device for their monitored medication and they were instructed to take medication from the EAM device whenever they took a "home" dose (e.g., evening or weekend doses). We used a second EAM device to monitor observed doses delivered at the OTP. For observed doses, the research assistant removed a dose from the EAM device and gave it to the participant to take (in addition to other medications used in the regimen). In subjects assigned to DAART, data from the "home" and "OTP" EAM devices will be combined for analyses.

### Power and Planned Statistical Analyses

The primary study outcome is suppression of HIV RNA < 50 copies/mL. We hypothesize that DAART will increase rates of viral suppression compared to SAT. At study outset, we planned to enroll 200 participants to provide 80% power to detect a 20% difference in HIV RNA suppression between the study arms at 12 months. However, enrollment was slower than anticipated because limited numbers subjects met the eligibility criteria. When addition of new study sites failed to fully rectify slow recruitment, we revised our primary outcome to viral suppression at three time points during active intervention (3, 6, and 12 months), rather than at a single time point (12 months). Using a repeated measures analytic approach, we calculated that a sample size of 120, assuming 15% loss to follow-up (i.e. effective sample size of 50 in each arm) and an intra-subject correlation of 0.2, will provide greater than 84% power to detect a 20% average difference in viral load suppression between the arms, given SAT viral suppression rates between 25% and 40%.

Secondary outcomes include HIV RNA < 400 copies/mL, change in log_10 _HIV RNA, change in CD4 cell count, cumulative use of ART, retention to the OTP, and acquisition of new antiretroviral resistance mutations. We will also evaluate differences in viral suppression at 18 months (6 months following the conclusion of the intervention) to assess for persistence of interventional effects. Finally, we will compare EAM in the study arms. We will use chi-squared and Wilcoxon rank sum tests to compare categorical and continuous variables, respectively. We will use mixed effects logistic and linear models to assess longitudinal outcomes. The primary analysis of viral suppression will be intent-to-treat, with missing values excluded. In sensitivity analyses, we will consider missing values to be failures and construct bounds for the potential bias.

### Participant Enrollment and Disposition

**Figure 1:**
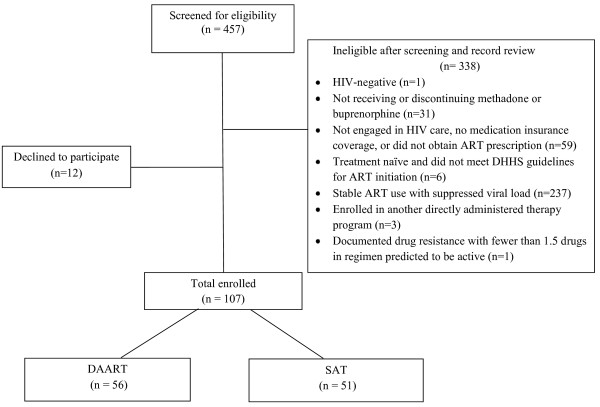
**Disposition of individuals screened for a trial comparing directly administered antiretroviral therapy to self-administered therapy in opioid treatment programs, Baltimore, Maryland**. ART, antiretroviral therapy; DHHS, Department of Health and Human Services; DAART, directly administered antiretroviral therapy; SAT, self-administered therapy.

A total of 457 individuals were screened for the study (Figure 1.). Of these, 338 were ineligible after screening and record review and 12 declined to participate. The most common reason for ineligibility was current ART use with suppressed viral load (n = 237), followed by disengagement in HIV care, lack of medication insurance, or failure to obtain an ART prescription (n = 59). A total of 107 participants were enrolled, with 51 assigned to SAT and 56 assigned to DAART. Compared to participants enrolled to the trial, the 12 individuals who declined to participate were more likely to be from site 4 and less likely to be African American (Table). Joiners and non-joiners were similar with respect to sex and age.

### Study sample characteristics

A median of 14 participants were enrolled at each of the five participating OTPs (range 8 to 39). Study participants were predominantly African American, approximately half were women, and the median age was 47 years (Table 1.). Fifty two percent of subjects had completed high school or a general equivalency diploma. Only 15% of participants were employed. Participants had received medication-assisted therapy at the OTP for a median of 11 months prior to enrollment, with a median methadone dose of 90 mg or a median buprenorphine dose of 19 mg. Forty percent and 20% were urine screen positive for cocaine and opiates, respectively, at study enrollment.

**Table 1:** Baseline characteristics of HIV-infected participants enrolled in a randomized trial comparing directly administered antiretroviral therapy to self administered therapy in opioid treatment programs, Baltimore, Maryland.

Characteristic	**Enrolled in study**^**a **^**(n = 107)**	**Declined to participate**^**a **^**(n = 12)**
Opioid treatment program (enrollment site)		
1	39 (36)	2 (17)
2	35 (33)	1 (8)
3	8 (8)	0
4	14 (13)	9 (75)
5	11 (10)	0
Female	51 (48)	7 (58)
Race		
African American	88 (82)	7 (58)
Caucasian/other	19 (18)	5 (42)
Age, years	47 (41-51)	46 (38-51)
Education		
Less than high school graduate	51 (48)	
High school graduate or equivalent	36 (34)	
Any college, n (%)	20 (19)	
Marital status		
Married or live with partner	17 (16)	
Never married	54 (51)	
Widowed	9 (8)	
Divorced or separated	27 (25)	
Considers himself/herself to be homeless	32 (30)	
Housing/living situation		
Own a house	3 (3)	
Rent an apartment/house	49 (46)	
Stay with family or friends	35 (33)	
Residential drug treatment	5 (5)	
Other residential facility or institution	11 (10)	
Live on street or in single room occupancy hotel	4 (4)	
Employed	16 (15)	
Duration of methadone or buprenorphine treatment, months	11 (2-49)	
Receiving methadone	99 (93)	
Methadone dose, mg	90 (70-120)	
Receiving buprenorphine	8 (7)	
Buprenorphine dose, mg	19 (14-27)	
Depression score^b^	11 (5-15)	
Urine drug test, positive results		
Cocaine	42 (40)^c^	
Benzodiazepine	14 (13)^c^	
Opiate (morphine/codeine)	21 (20)^c^	
Oxycodone	1 (1)^c^	
Nadir CD4 count, cells/mm^3^	102 (30-218)	
Current CD4 count, cells/mm^3^	182 (69-309)	
HIV RNA log_10 _copies/mL	4.7 (4.0-5.1)	
Hospitalized in prior 3 months	34 (32)	
Prior AIDS-defining opportunistic condition	66 (62)	
Hepatitis C antibody positive	87 (81)	
Antiretroviral naive	21 (20)	
Category of prescribed antiretroviral regimen		
PI + NRTIs	83 (78)	
NNRTI + NRTIs	16 (15)	
NRTIs only	1 (1)	
Other	7 (7)	
Drug classes included in antiretroviral regimen (not mutually exclusive)		
NRTI	97 (91)	
Ritonavir-boosted PI	85 (79)	
PI (not boosted with ritonavir)	4 (4)	
NNRTI	21 (20)	
Integrase inhibitor	7 (7)	
Dosing frequency of prescribed antiretroviral regimen		
Once daily	65 (61)	
Twice daily	42 (39)	

PI, protease inhibitor; NRTI, nucleoside reverse transcriptase inhibitor; NNRTI, non-nucleoside reverse transcriptase inhibitor.

^a ^Values are frequency (%) or median (interquartile range)

^b ^Center for Epidemiologic Studies Depression (CES-D) Scale (7). Higher values indicate more numerous or more severe depressive symptoms (range 0 to 30).

^c ^From 106 subjects.

In general, study participants had advanced HIV disease, 62% had a history of Centers for Disease Control and Prevention Category C opportunistic conditions [17], the median nadir CD4 count was 102 cells/mm^3^, the median current CD4 was 207 cells/mm^3^, and the median HIV RNA levels was 4.7 log_10 _copies/mL. Twenty percent of subjects were ART naïve at enrollment, and the prescribed regimen was dosed once daily in 61% and twice daily in 39%. Nearly 80% of participants were treated with protease inhibitor-based regimens, the majority of which were ritonavir-boosted.

## Discussion

This study is designed to assess the efficacy of DAART compared to SAT among HIV-infected subjects attending OTPs for maintenance methadone or buprenorphine therapy, and builds upon our developmental work with DAART in this setting [7,18]. Our study is the second randomized controlled trial of DAART in OTPs. A recent study from the Bronx, New York, found that OTP-based DAART was associated with statistically significantly higher rates of adherence and viral suppression than SAT [6]. OTPs are attractive settings to deliver DAART because HIV-infected drug users are at higher risk for non-adherence [19] and suboptimal ART outcomes [20] compared to other HIV risk groups, and OTP regulations necessitate frequent contact with patients.

To date, trials of DAART have yielded mixed findings regarding efficacy [2,3]. However, completed trials have varied considerably in setting, population studied, and method of delivering DAART. For example, trials have been conducted in resource-rich [6,21-24] or resource-poor settings [25-28], enrolled predominantly ART-naïve [24] or ART-experienced participants [6,22,23], enrolled unselected participants [21,24,25,27,28] or participants with adherence barriers [6,22,23], and used healthcare workers [21-23] or unpaid peers (often family or friends) [6,24-28] to deliver DAART. Additionally, trials have differed in methodological factors including sample size, duration of intervention, primary outcome, method of measuring adherence, and follow-up after completion of the DAART intervention.

Our study has several strengths. First, our target population is HIV-infected drug users (most with demonstrated non-adherence in previous treatment attempts). Of completed DAART trials, those targeting individuals with documented non-adherence, active drug use, or other adherence barriers have been more likely to show efficacy compared to studies enrolling primarily unselected and ART-naïve participants[2,3,29,30]. Moreover, labor intensive adherence interventions are most likely to be cost-effective when delivered to those at highest risk of poor outcomes [31]. Second, ours is one of the only trials to assess the efficacy of DAART over 12 months. Two other studies that assessed DAART interventions for 12 months were conducted in sub-Saharan Africa and used peers to observe dosing [25,28]. Third, similar to other studies [24-27,32], our study will assess the persistence of any treatment arm differences that emerge during the intervention period at a post-intervention follow-up visit. Fourth, as has been done in other studies [33-35], we will assess the effect of DAART on the acquisition of new drug resistance mutations. Finally, ours will be among the few studies to measure adherence with EAM in both the DAART and SAT arms [24]. In the DAART arm, EAM will be done for both observed doses at the OTP and for self-administered doses taken on nights and weekends. A study conducted in prisons found that EAM for doses 'observed' by medical staff was lower than that documented in medication administration records [36], highlighting the importance of using consistent measurement techniques for self-administered and "observed" dosing.

An important limitation of our study is the relatively small sample size, which limits our ability to detect small, but potentially clinically meaningful, differences between study conditions. Slow enrollment to our study was multifactorial. We encountered lower prevalences of HIV at the OTPs than expected, although pre-study estimates were based on limited data. Additionally, once we had screened the population at an OTP, we were dependent on slot turnover for new candidates. As can be seen in the Figure, the predominant reason for exclusion was stable ART use with suppressed viral load, which accounted for 70% of exclusions. This may be a positive finding that reflects improving access to ART among HIV-infected drug users during the study period compared to the late 1990s [37]. One way to address slow enrollment would have been to include participants who were taking ART and had suppressed viral loads, as has been done in other studies [6,23]. However, we chose not to enroll such participants because we were concerned that the intervention could have heterogeneous effects in individuals who were failing (or not taking) therapy and those who were doing well with treatment. Consequently, our study population is restricted to those participants most in need of intervention - those not taking ART when indicated or experiencing virologic failure on ART.

The second most common reason for exclusion was disengagement from HIV care, absence of active insurance coverage, or failure to obtain ART prescription, which accounted for 59 exclusions. We considered such individuals good candidates for the trial and made efforts to assist them by scheduling appointments with HIV providers, providing appointment reminders, and assisting with paperwork. However, particularly at sites where on-site HIV care was not available, such efforts were often unsuccessful. This highlights the importance of addressing basic access and engagement to care in trials of medication adherence interventions. In general, willingness to join the study was not a substantial barrier to recruitment, with only 12 individuals declining to participate. The majority (9) of these subjects were from a single site, which was the only site that did not have on-site HIV or medical care. Anecdotally, individuals at this site were more apt to cite confidentiality concerns as barriers to joining than individuals at other sites.

In summary, we recently completed enrollment for a randomized controlled trial comparing DAART to SAT among HIV-infected participants attending OTPs. Important clinical questions remain about whether DAART is a useful adherence strategy, and if so, in which populations and under what implementation models. Our study will contribute to existing data on this topic and guide future approaches.

## Competing interests

The authors declare that they have no competing interests.

## Authors' contributions

BAM developed and implemented the study protocol, oversaw research assistants, and wrote the initial draft of the manuscript; KC assisted in the development of study protocols and managed study data, RDM provide guidance on study design and methods, CR provided guidance on study design and methods and provided expertise on electronic adherence monitoring, NG conducted power analyses and planned statistical analyses, MEM provided input on study design and methods and provided access to participants, SG provided guidance on protocols and recruitment methods and provided access to study participants, KKO provided guidance on protocols and recruitment methods and provided access to study participants, and GML obtained funding for the study, oversaw its conduct, and completed the final manuscript version. All authors have read, contributed to, and approved the manuscript.

## Pre-publication history

The pre-publication history for this paper can be accessed here:

http://www.biomedcentral.com/1471-2334/11/45/prepub
